# Phytochemical Profiles, Antioxidant and Antibacterial Activities of Grape (*Vitis vinifera* L.) Seeds and Skin from Organic and Conventional Vineyards

**DOI:** 10.3390/plants9111470

**Published:** 2020-10-30

**Authors:** Cristiana Radulescu, Lavinia Claudia Buruleanu, Cristina Mihaela Nicolescu, Radu Lucian Olteanu, Marius Bumbac, Georgeta Carmen Holban, Jesus Simal-Gandara

**Affiliations:** 1 Faculty of Sciences and Arts, Valahia University of Targoviste, 130004 Targoviste, Romania; 2 Institute of Multidisciplinary Research for Science and Technology, Valahia University of Targoviste, 130004 Targoviste, Romania; 3 Faculty of Environmental Engineering and Food Science, Valahia University of Targoviste, 130004 Targoviste, Romania; 4 Doctoral School, University of Agronomic Sciences and Veterinary Medicine of Bucharest, 011464 Bucharest, Romania; carmenholban@yahoo.com; 5 Nutrition and Bromatology Group, Department of Analytical Chemistry and Food Science, Faculty of Food Science and Technology, University of Vigo—Ourense Campus, E-32004 Ourense, Spain

**Keywords:** red grape extract, organic/conventional vineyard, phenolycs, flavonoids, antioxidant activity, antimicrobial activity, statistical analysis

## Abstract

The therapeutic benefits of extracts obtained from different red grape fractions were thoroughly studied, however, data regarding the comparison of phytochemical extracts prepared from the same varieties coming from organic *versus* conventional management systems are rather lacking. The present study aimed at comparing some of the phytochemical characteristics and antimicrobial activity of hydroalcoholic (50% *v/v*) extracts obtained from four varieties of red grapes cultivated respectively in organic and conventional vineyards. Total flavonoid content, total phenolic compounds, and antioxidant activity were determined by molecular absorption spectroscopy. Antimicrobial activity of the studied extracts was evaluated against common bacterial strains isolated from different habitats according to specific lab procedures. The analyses were performed in solid broths by applying the disk diffusion method, which allowed for the simultaneous determination of the spectrum of the sensitivity of the tested bacteria as well as the values of the minimum inhibition concentration (MIC). It was found that favorable antagonistic activities against the tested bacteria strains were exhibited by the hydroalcoholic extracts from the seeds of the organic varieties, respectively the skin of the conventional varieties.

## 1. Introduction

In the EU, 55 million tons of agricultural vegetal and forestry wood waste were produced in 2016 [1]. These residues could be transformed in bio-based products (e.g., feed, bio-pesticides, bio-plastics, etc.) or valorized as a source of added-value molecules [2,3,4]. The use of natural treatments and plant extracts dates from thousands of years. Despite the significant development of synthetic pharmaceuticals, in recent decades, the interest for herbal medicine and cosmetic formulations using various plant fractions and extracts has gradually increased [5,6,7]. Among the reasons behind these facts were the resistance of some microbes to existing antimicrobial agents as well as some specific unwanted side effects, and sometimes the high cost of the treatments.

The wine-making process generates significant volumes of by-products consisting of skins, seeds, and stalks in different proportions [8,9]. Application of circular economy principles in this domain determined an evolution of activities aiming at the re-use of these by-products with positive economic impacts [4]. For instance, Technavio, the global market research firm with significant industry expertise, has monitored the grape seed market in the past years and made corresponding forecasts. According to the report published in 2020, [10] the grape seed oil market is poised to grow by 73.8 million USD during 2020–2024, progressing at a compound annual growth rate (CAGR) of 4% during the forecasting period. This study was a detailed analysis of vendors operating in the grape seed market around the world, and together with information on current market scenarios, the latest trends and drivers, identified that the therapeutic use of these parts of grapes, mainly via cosmetic products, is the first reason leading the market environment in this field. New natural sources of active agents, mainly antioxidants and antimicrobials, incorporated in cosmetic and food products, have become a concern for many studies in the last few years [7,11,12,13]. In addition, selective extraction of some phytochemicals from natural sources is considered an opportunity to substitute synthetic chemicals that are currently in use in the cosmetic industry. On the other hand, once food biomass components are used, the added value is an appreciated benefit for food and pharmaceutical industries [14].

Flavonols, anthocyanidins, hydroxybenzoic acids, and stilbenes are the main phenolic compounds from grapes, occurring in the human diet [15]. They may be efficient in the treatment of acne and other minor skin problems, but also in the treatment of serious dermal diseases such as cancer. On the other hand, chemical compounds from grapes, in particular from skins and seeds that become the main components of the biomass, have resulted from the processes in the wine industry and offer valuable opportunities as cosmetic additives due to their availability and chemical diversity. The profile of bioactive compounds contained by the so-called natural cosmetic products may be different according to the characteristics of the extracts used in their fabrication processes [16,17,18,19,20,21,22].

In the last years, consumers have preferred cosmetics made of natural ingredients with active functions on their skin and would prefer to spend higher amounts of money for a cosmetic that promises skin benefits [23]. Topical administration and transport of bioactive ingredients through skin may follow various pathways, and their diffusion across different layers is influenced by the solubility, polarity, molecular weight, and other characteristics of these compounds [24,25,26].

The term “cosmeceuticals” has been used from the second half of the twentieth century [27,28] to define topical products with an effect on both skin appearance and functioning. These types of products are at the same time a “cosmetic” and a “pharmaceutical”, and have a lasting effect through physiological and/or pharmacological action [29,30,31].

Organic versus conventional vineyard management is discussed in the literature to compare their sustainability performance [32] including the analysis of their impact on the human resource to assess the potentially toxic elements in the soil-plant-air system [33], in order to report environmental and health risks for field workers and grape consumers to evaluate their impact on biodiversity [34] or to substantiate the influence of the agricultural practices on grape yeast succession and wine quality [35]. Other studies are focused on the decay extension and the nutritional quality of organic and conventionally grown table grapes [36] and also on the characteristics of organic wines versus conventional wines [37]. Previous research has studied the antibacterial activity of grape tissue extracts [38] or the phytochemical composition [39]. However, few studies have assessed both the chemical characteristics and the antimicrobial activity of grapes/grape berry tissue from organic and conventional vineyards. Moreover, the studied grape varieties, the geographical area of management, and the differences in vineyard management practices may affect the potential of certain grape extracts to be used in different formulation in the pharmaceutics or food industry. Thus, the present study aims to evaluate/explore the potential of four grapes varieties cultivated in Romania in an organic vineyard, and respectively in a conventional one. The hydroalcoholic extracts obtained from grape berry tissues (skin and seeds) were characterized to determine total flavonoid content, total polyphenolic content, and antioxidant activity. The antimicrobial activity of the extracts was tested against bacteria strains isolated from natural habitats. The statistical analysis of data revealed that relationships between the chemical composition and the antioxidant and antibacterial activities were different according to the berry tissue, the vineyard management, and the grape variety.

## 2. Results

### 2.1. Phytochemical Characterization of the Extracts

Table 1 presents the phytochemical characterization of grape skin and seed extracts for different varieties of grapes harvested from the organic and conventional vineyard. The hydroalcoholic extracts obtained from dried seeds had higher values for total phenolic content (TPC), total flavonoid content (TFC), and antioxidant activity (AA) (TPC, TFC, and AA compared with the extracts obtained from grape skins, regardless of the grape treatment method. In the case of grape skin extracts, it was found for all varieties of grapes that the organic way of treating the vineyards leads to close phytochemical characteristics compared to the characteristics of grapes from the conventional treated vineyard.

Furthermore, when comparing Merlot, Feteasca Neagra, and Pinot Noir dry seed extracts, notable differences were observed between the phytochemical characteristics of the grape varieties harvested from the organic vineyards compared to the varieties from the vineyards conventionally treated. The hydroalcoholic extracts of seeds harvested from the organic vineyard had higher values for TPC, TFC, and AA. On the other hand, the Muscat Hamburg variety had similar values for the phytochemical characteristic of the grape seed extracts, if we compare the grapes that come from the organic vineyard and the one treated with conventional treatments.

The ANOVA test applied to the data from Table 1 revealed that the content of phytochemicals and the antioxidant activity of the four grapes varieties were different (*p* < 0.05). From the quantitative point of view, the vineyard management affected (*p* < 0.05) the total phenolic content, the total flavonoid content, and the antioxidant activity of the grapes’ hydroalcoholic extracts.

The technique of multiple comparisons (the post-hoc analysis) emphasized the groups differing as average. Thus, the grape varieties differ from each other concerning the antioxidant activity of the hydroalcoholic extracts obtained from skin (*p* < 0.05). Regardless of the anatomic part (skin/seeds) used for extraction, vineyard management significantly affected (*p* < 0.05) only the total phenolic content of the extracts, while the total flavonoid content and the antioxidant activity of extracts were not influenced (*p* > 0.05) by vineyard management. The grape variety significantly affected (*p* < 0.05) the total phenolic content of extracts, especially for organic vineyard management in the case of both grape berry tissue extracts. Highest values of TPC were recorded for hydroalcoholic extracts of Feteasca Neagra (i.e., grape berry skin) and Pinot Noir (i.e., grape berry seeds) varieties.

### 2.2. Antibacterial Activity of the Grapes Extracts

**Figure 1:**
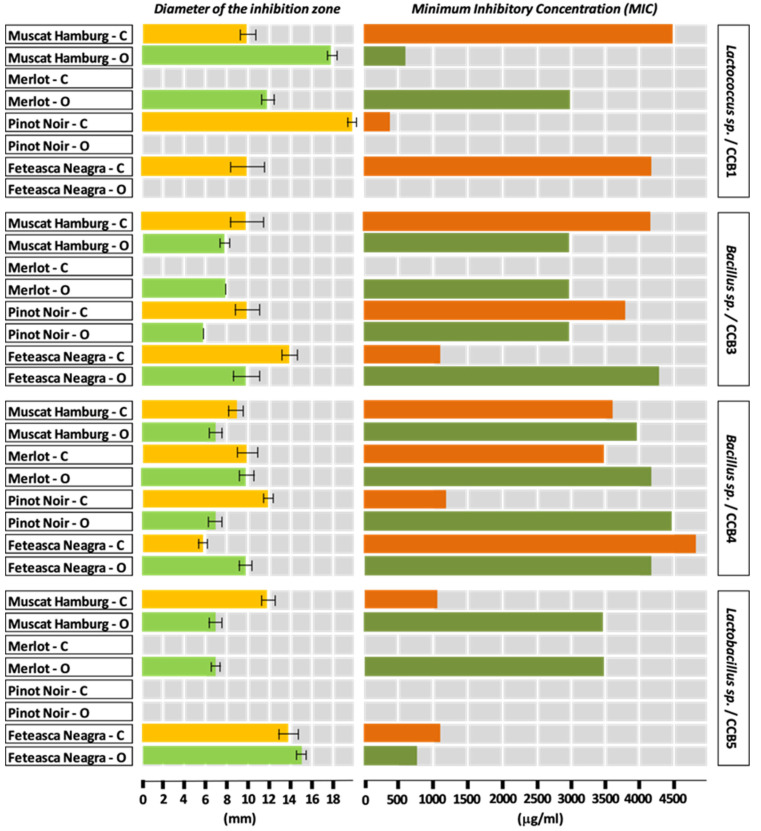
Diameter of the inhibition zone (means ± standard deviation) and minimum inhibitory concentration for hydroalcoholic red grape skin extracts from conventional (C) and organic (O) vineyards, based on the CCB1, CCB3, CCB4, and CCB5 bacterial strains.

**Figure 2:**
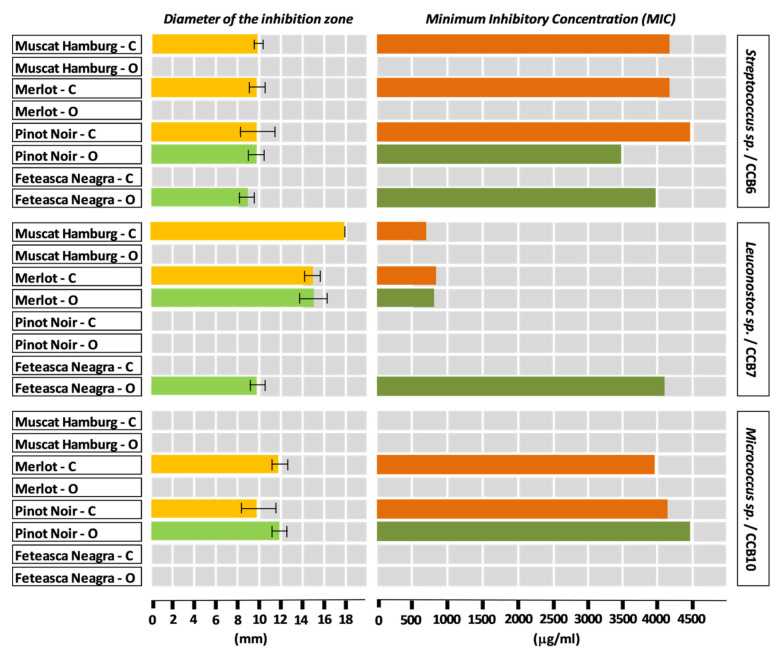
Diameter of the inhibition zone (means ± standard deviation) and minimum inhibitory concentration for hydroalcoholic red grape skin extracts from conventional (C) and organic (O) vineyards, based on CCB6, CCB7, and CCB10 bacterial strains.

Most hydroalcoholic extracts from grapes (skin and seeds) showed antimicrobial action against the tested bacterial strains. The antibacterial effect of the extracts was expressed relative to against a certain strain. The diameters of the inhibition zones of the bacterial growth by hydroalcoholic extracts from grapes skin are presented in Figure 1 and Figure 2, the majority being within the range of 6–14 mm.

The diameters of the areas of growth inhibition by the hydroalcoholic extracts from skin of the grapes varieties cultivated in the conventional system varied as follows: Pinot Noir (CCB1, 20 mm) > Muscat Hamburg (CCB7, 18 mm) > Merlot (CCB7, 15 mm) > Feteasca Neagra (CCB3, CCB5, 14 mm) > Muscat Hamburg (CCB5, 12 mm), Merlot (CCB10, 12 mm) and Pinot Noir (CCB4, 12mm). An average value of 10 mm was determined for the following varieties and strains of bacteria: Muscat Hamburg (CCB1, CCB3 and CCB6), Merlot (CCB4 and CCB6), Pinot Noir (CCB3, CCB6 and CCB10), and Feteasca Neagra (CCB1), respectively.

The hydroalcoholic extract from the skin of Muscat Hamburg grapes cultivated in the conventional system proved to have a broad spectrum of antibacterial activity.

The antibacterial activity of the analyzed grape varieties cultivated in the conventional system varied as follows: Muscat Hamburg (active against six of the eight bacterial strains) > Pinot Noir (active against five of the eight bacterial strains) > Merlot (active against four of the eight bacterial strains) > Feteasca Neagra (active against three of the eight bacterial strains). Referring to the tested bacterial strains, the results showed an increased sensitivity to the antimicrobial compounds of the hydroalcoholic extracts from the skin of these varieties generally manifested by the strains CCB4, CCB1, CCB3, and CCB6, followed by the strains CCB5, CCB7, and CCB10. It should be noted that the CCB11 strain did not show sensitivity to any of the tested hydroalcoholic extracts from skin (data not shown).

The diameters of the areas of growth inhibition by the hydroalcoholic extracts from skin of the grape varieties cultivated in the organic system varied as follows: Muscat Hamburg (CCB1, 18 mm) > Merlot (CCB7, 15 mm) and Feteasca Neagra (CCB5, 15 mm) > Merlot (CCB1, 12 mm). An average value of 10 mm was determined for the following varieties and strains of bacteria: Merlot (CCB3), Pinot Noir (CCB5), and Feteasca Neagra (T2, CCB3, and CCB6), respectively.

The antibacterial activity of the analyzed grape varieties cultivated in the organic system varied as follows: Merlot and Feteasca Neagra (active against five of the eight bacterial strains) > Muscat Hamburg and Pinot Noir (active against four of the eight bacterial strains). Taking into account the antibacterial spectrum of the chemical compounds from the skin of these grape varieties, the results indicated a sensitivity of the strains CCB3 and CCB4 (in the case of all four extracts), for the rest of the strains a differentiation depending on the grape variety being observed (CCB5—three of the four extracts; CCB1, CCB6, CCB7—two of the four extracts; CCB10—one of the four extracts). Similar to the situation mentioned in the case of the grape varieties cultivated in the conventional system, the CCB11 strain did not show sensitivity to any of the tested hydroalcoholic extracts from the skin of the organic varieties (data not shown).

Regarding the system of management, the sensitivity of the tested bacterial strains to the chemical compounds from the grape skin extracts was nearly similar, the observed differences being associated with specific bacterial strains: CCB4 and CCB3—strains sensitive to all or almost all extracts; CCB1, CCB5, and CCB6—strains sensitive to most extracts; CCB7 and CCB10—strains sensitive to about half of the number of the analyzed hydroalcoholic extracts from skin.

The comparative analysis of the data from Figure 1 and Figure 2 highlights that the grape varieties cultivated in the conventional system showed antibacterial activity against a smaller number of bacterial strains as against the same grape varieties cultivated in the organic system. By reporting to the values of the diameters of the zones of inhibition of bacterial growth, the hydroalcoholic extracts from the skin of the grape varieties cultivated in the conventional system proved to be more efficient compared to the extracts from the organic grape varieties.

The MIC values of the grape skin extracts ranged from 420 to 4500 µg/mL (Figure 1 and Figure 2), emphasizing the antagonistic activity of these extracts toward most of the tested bacterial strains.

**Figure 3:**
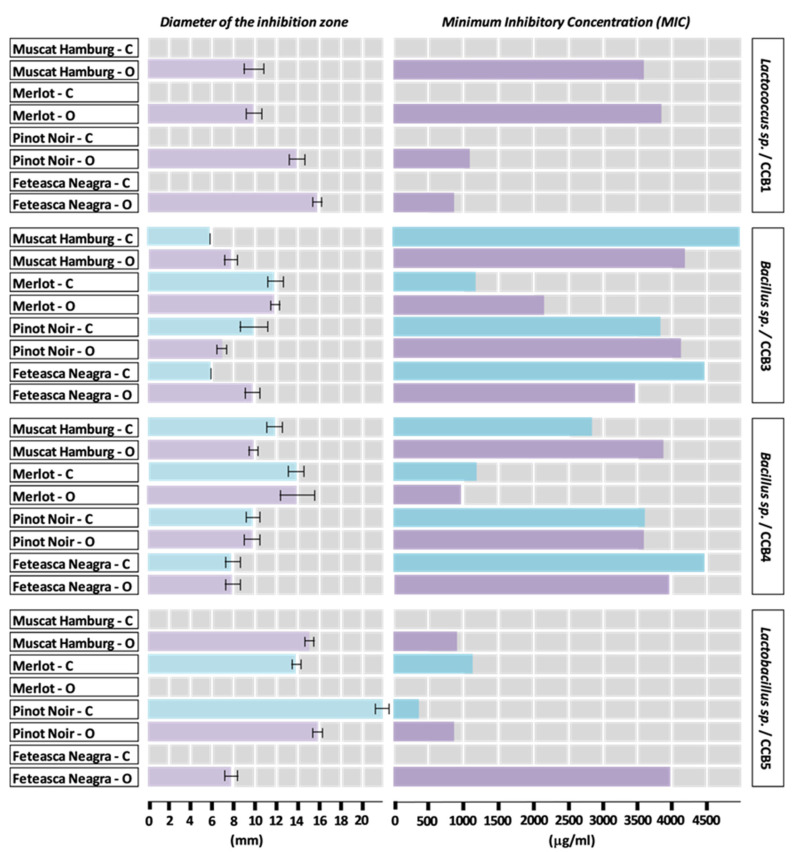
Diameter of the inhibition zone (means ± standard deviation) and minimum inhibitory concentration for hydroalcoholic red grape seed extracts from conventional (C) and organic (O) vineyards, based on CCB1, CCB3, CCB4, and CCB5 bacterial strains.

**Figure 4:**
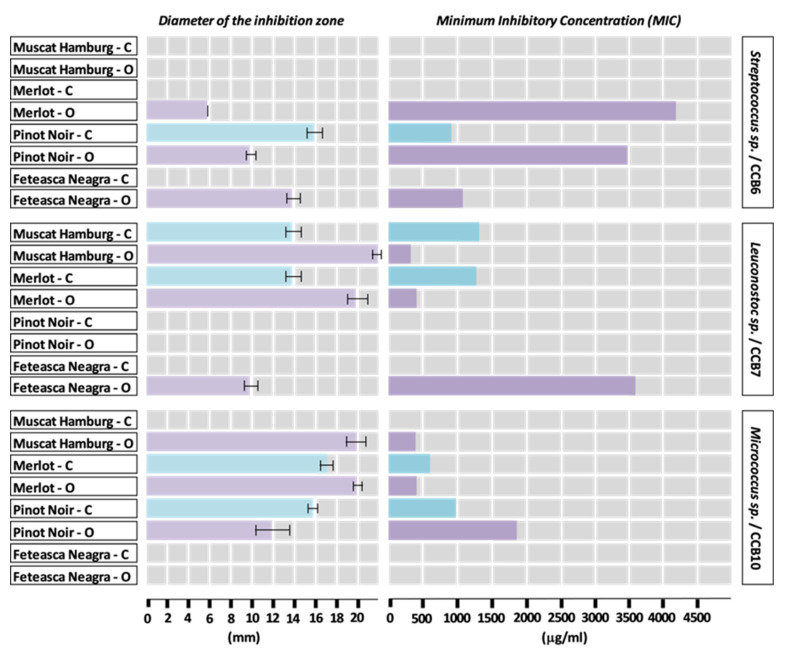
Diameter of the inhibition zone (means ± standard deviation) and minimum inhibitory concentration for hydroalcoholic red grape seed extracts from conventional (C) and organic (O) vineyards, based on CCB6, CCB7, and CCB10 bacterial strains.

The diameters of the inhibition zones of the bacterial growth by hydroalcoholic extracts from grapes seeds are presented in Figure 3 and Figure 4.

The diameters of the areas of growth inhibition by the hydroalcoholic extracts from seeds of the grapes varieties cultivated in the conventional system varied as follows: Pinot Noir (CCB5, 22 mm) > Merlot (CCB10, 17 mm) > Pinot Noir (CCB6 and CCB10, 16 mm) > Muscat Hamburg (CCB7, 14 mm), Merlot (CCB4, CCB5, CCB7, 14 mm) > Muscat Hamburg (CCB4, 12 mm), Merlot (CCB3, 12 mm) > Pinot Noir (CCB3 and CCB4, 10 mm). Minimum values of 6 mm were determined for Muscat Hamburg and Feteasca Neagra, both seed extracts showing weak activity against the same bacterial strain (CCB3).

The values of the diameters of the areas of growth inhibition suggest a generally higher sensitivity to Merlot and Pinot Noir seed extracts (active against five of the eight strains studied) compared to the Muscat Hamburg and Feteasca Neagra varieties (active against three and two, respectively, of the eight bacterial strains studied). Referring to the tested bacterial strains, the results showed an increased sensitivity to the antimicrobial compounds of the hydroalcoholic extracts from the seeds of these varieties cultivated in a conventional system manifested by the strains CCB3 and CCB4 (sensitive to all the four extracts analyzed), followed by the strains CCB5, CCB7, and CCB10. It should be noted that the strains CCB1 and CCB11 (data not shown) did not show sensitivity to any of the tested hydroalcoholic extracts from seeds.

The diameters of the areas of growth inhibition by the hydroalcoholic extracts from seeds of the grape varieties cultivated in the organic system varied as follows: Muscat Hamburg (CCB7, 22 mm) > Muscat Hamburg (CCB10, 20 mm) and Merlot (CCB7 and CCB10, 20 mm) > Pinot Noir (CCB5, 16 mm) and Feteasca Neagra (CCB1, 16 mm) > Muscat Hamburg (CCB5, 15 mm) > Merlot (CCB4, 14 mm), Pinot Noir (CCB1, 14 mm), and Feteasca Neagra (CCB6, 14 mm).

The hydroalcoholic extracts obtained from the seeds of the organic varieties of Muscat Hamburg and Merlot showed a strong antagonistic activity against the bacterial strains CCB7 and CCB10, with MIC values varying between 380 and 450 µg/mL.

The values of the diameter of the area of inhibition of bacterial growth also indicated that the tested bacterial strains were generally sensitive to the chemical compounds of the seed extracts obtained from grapes cultivated in the organic system. The antibacterial activity of extracts, manifested against six of the eight strains, was relatively high. CCB1, CCB3, and CCB4 strains were sensitive to all four extracts, while for the other strains, a differentiation was observed according to variety (CCB5, CCB6, CCB7, and CCB10 were sensitive to three of the four extracts). The CCB11 strain did not show sensitivity to any of the tested hydroalcoholic extracts from the seeds of the organic varieties (data not shown).

Referring to the hydroalcoholic extracts from seeds, an average value of 10 mm of the diameter of the area of inhibition of bacterial growth was determined for the following varieties and strains of bacteria, respectively: Pinot Noir cultivated in conventional system (CCB3, CCB4), Muscat Hamburg (CCB1, CCB4), Merlot (CCB1), Pinot Noir (CCB4, CCB6), and Feteasca Neagra (CCB3, CCB7)—all cultivated in the organic system.

The seed extracts from the Feteasca Neagra variety cultivated in the conventional system showed low antibacterial activity and only against the CCB3 (6 mm) and CCB4 (8 mm) strains.

The standardization of the hydroalcoholic extracts obtained from the skin and the seeds of the grapes and the knowledge of their corresponding MIC values are of particular practical importance, in order to develop applications for different fields, applications in the frame of which the bacteriostatic and the bactericide activity are scientifically substantiated in relationship with a certain species of microorganisms.

An overview of the data related to the antibacterial effects of the extracts obtained from the skin and seeds of the grapes cultivated in both systems (conventional and organic) revealed preliminary conclusions, which can be used as a starting point in order to correlate the antimicrobial activity of grapes with their chemical composition.

Thus, regardless of the anatomical part of the grapes, the 16 extracts showed antibacterial activity against the CCB4 strain and with only the Merlot variety cultivated in a conventional vineyard against the CCB3 strain as an exception. The antibacterial activity of all extracts was null relative to the CCB11 strain.

The most effective antibacterial spectrum was determined in the case of the hydroalcoholic extracts from seeds of organic grown varieties, against the CCB7 strain: Muscat Hamburg (22 mm) and Merlot (20 mm). The conventional cultivated Pinot Noir variety also showed a remarkable antibacterial activity in relation to the CCB1 strain (skin extract, 20 mm), respectively, the CCB5 strain (seeds extract, 22 mm).

Discussing the data in relationship with the grape varieties, a noticeable differentiation of the antibacterial activity depending on the culture system was observed in the case of the Muscat Hamburg. Thus, the most active extracts were those obtained from the skin of the grapes cultivated in the conventional system, respectively, from the seeds of the same variety cultivated in the organic system. For the Merlot variety, the hydroalcoholic seed extracts showed a significant antibacterial activity against the tested strains, regardless of the system of management. A relatively high antibacterial activity was observed in the case of the seed extracts obtained from the Pinot Noir and Feteasca Neagra varieties cultivated in the organic system.

The hydroalcoholic extracts from the skin of the grapes grown in the conventional system showed efficiency against the growth of bacteria compared to extracts from grapes grown in the organic system. Analyzing the data referring to the extracts obtained from grape seeds, a significant increased antibacterial activity was determined for varieties grown in organic vineyards. The resistance of the grape varieties to different environmental factors (including microbial attack) could explain the antibacterial activity of extracts obtained from seeds (organic varieties) and skins (conventional varieties), respectively.

### 2.3. Statistical Analysis of Data

#### 2.3.1. Correlations of the Phytochemical Parameters and the Antibacterial Activity of the Bacterial Strains

To analyze the relationship between the different variables quantified, the Pearson correlation was applied. A bivariate strong correlation (*p* < 0.01) characterized the pairs: TPC-TFC (*r* = 0.842), AA-TPC (*r* = 0.9), AA-TFC (*r* = 0.824). Low correlations were established for the following pairs: AA-AA against the CCB4 strain (*r* = 0.384), AA-AA against the CCB4 strain (*r* = 0.387), TFC-AA against the CCB4 strain (*r* = 0.243), and TPC-AA against the CCB4 strain (*r* = 0.243). For the other bacterial strains whose resistances to the chemical compounds of the hydroalcoholic extracts obtained from skins and seeds was established, the relationship between the antimicrobial activity and the phytochemical parameters was weak (i.e., *r* = 0.284 for the pair TFC-AA against the CCB10 strain) or inexistent (i.e., *r* = 0.001 for the pair TFC-AA against the CCB3 strain). Although relatively higher values of the diameters of inhibition were obtained, for example, against the CCB4 strain, regardless of the type of extract used, Pearson values highlighted that this behavior was independent of the flavonoid and phenolic contents and the antioxidant activity, respectively. 

**Figure 5:**
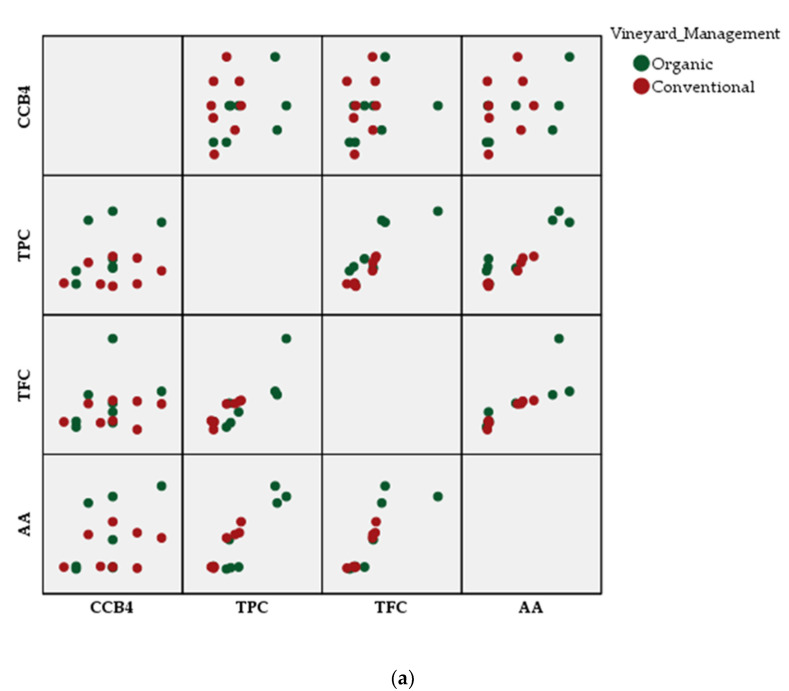

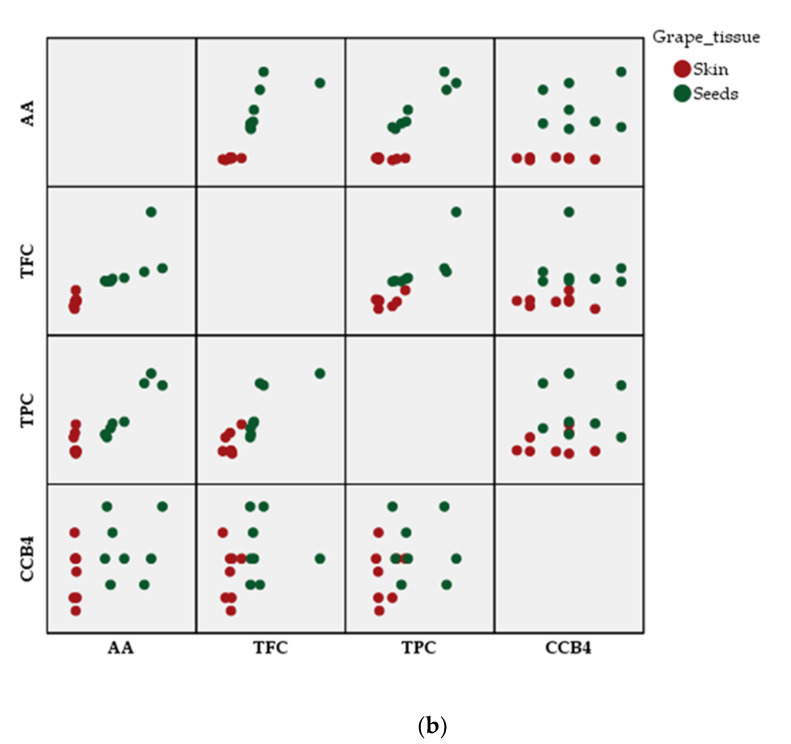
Correlations between the phytochemical parameters of the grape extracts and their antimicrobial activity, depending on: (**a**) the management system; (**b**) the anatomic part of the plant.

Figure 5 shows the correlation between TPC, TFC, AA, and antibacterial activity of the extracts against CCB4 strain, based on both management system (a) and grape berry tissue (b). The graphical representation reveals that most of the organic varieties and the seeds of the grapes are sources to be exploited in terms of compounds with antioxidant activity.

Degree of correlation between the management system and the antioxidant activity, respectively, the content of total flavonoids determined in hydroalcoholic extracts can be considered inexistent (Table 2). In contrast, the Eta squared test showed that the anatomic part of the grapes from which the extracts were obtained, also accounting much in rating the concentration of the antioxidant compounds and the antioxidant activity of the analyzed extracts. A low value of Eta squared of 0.241, as a measure of the association between total phenolic content and management system of the grapes, was obtained.

Regression analysis was conducted between the dependent variables (flavonoid and phenolic contents and antioxidant activity of the hydroalcoholic extracts) and grape varieties, vineyard management, and the anatomic parts of the grapes as independent variables. The equations of regression and the associated values of R square are shown in Table 3.

A strong relationship between all the analyzed variables was established for the predicted antioxidant activity of the extracts (R square = 0.82). The values of the total phenolic content were also in a relatively high proportion correlated with the dependent variables, while only 56% from the variation of the total flavonoid content was explained by the grape variety, vineyard management (organic/conventional), and anatomic part of the grapes.

Values of Eta squared, as a measure of association between the antimicrobial activity of extracts against the bacterial strains tested and the management system of the grapes, respectively, their anatomic part emphasized that the correlations were rather non-existent (Table 4).

**Figure 6:**
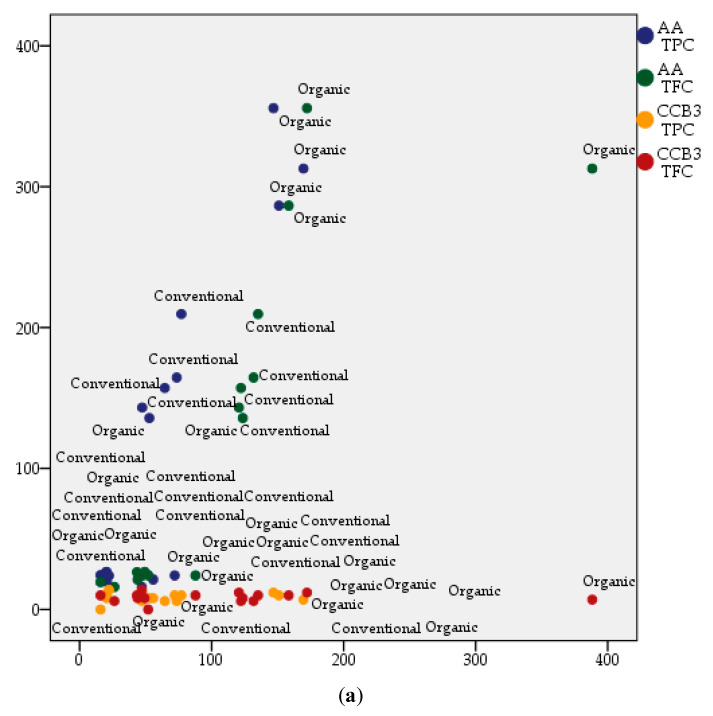

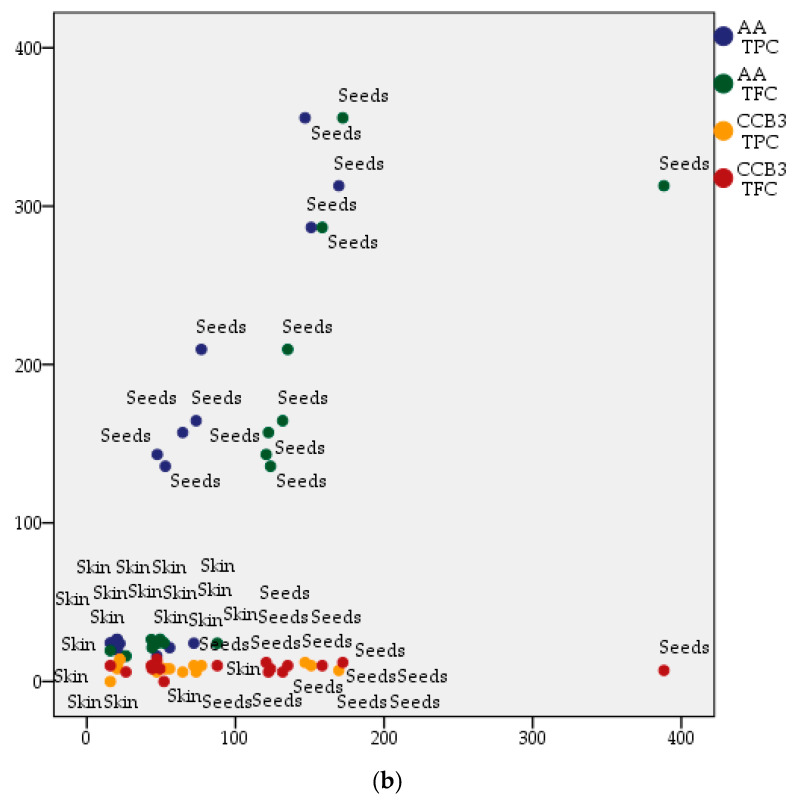
Multiple correlations between the phytochemical parameters and antimicrobial activity of the extracts, depending on: (**a**) the management system; (**b**) the anatomic part of the grapes.

The lack of correlations was also emphasized if more pairs regarding the phytochemicals and the antimicrobial activity of extracts were analyzed, considering the system of grape management and the anatomic part of the grapes (Figure 6).

#### 2.3.2. Statistical Package for the Social Sciences SPSS Classification: Hierarchical Cluster Analysis

**Figure 7:**
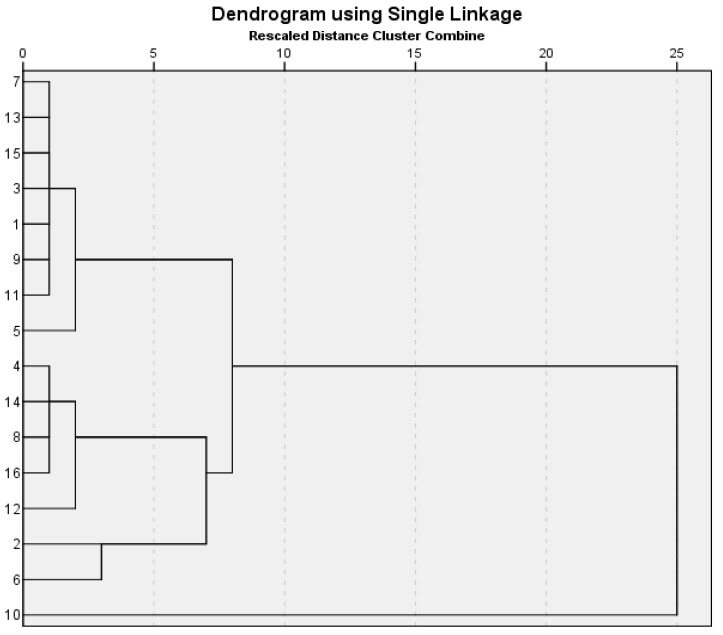
Dendogram of the variables of interest. Merlot extracts: (**1**) Organic skin; (**2**) Organic seeds; (**3**) Conventional skin; (**4**) Conventional seeds. Feteasca Neagra extracts: (**5**) Organic skin; (**6**) Organic seeds; (**7**) Conventional skin; (**8**) Conventional seeds. Pinot Noir extracts: (**9**) Organic skin; (**10**) Organic seeds; (**11**) Conventional skin; (**12**) Conventional seeds. Muscat Hamburg extracts: (**13**) Organic skin; (**14**) Organic seeds; (**15**) Conventional skin; (**16**) Conventional seeds.

Hierarchical cluster analysis applies to small sets of data. In the present study, the question arose as to whether there were identifiable groups in the set of variables, with similar characteristics, that characterize grape varieties (total phenolics content, total flavonoids content, antioxidant activity of the hydroalcoholic extracts from skin and seeds, respectively, on their antimicrobial activity against certain bacterial strains isolated from natural habitats). The square of the Euclidean distance (as a measure of distance) was used in order to construct the matrix of similarities. The nearest neighbor method of aggregation was applied to form clusters considering all the analyzed cases. Figure 7 illustrates the clustering agglomeration. All grape varieties with similar characteristics (in terms of variables of interest mentioned above) together form clusters.

According to the phytochemical characteristics and the antibacterial activity of the extracts against the tested strains, in the initial stage of agglomeration, different grapes varieties together formed two clusters (Figure 7). Feteasca Neagra (organic skin) and Pinot Noir (conventional seeds) were involved in the second stage of clustering, while Muscat Hamburg (conventional seeds) remained isolated until the end stage. In the third stage were involved the Merlot organic seeds and Feteasca Neagra organic seeds. Finally, the clustering method leads, depending on the grape variety and their anatomical part, to the formation of two clusters clearly defined.

All extracts obtained from grape skins, regardless of the system of management, aggregated in the same cluster, except for one belonging to the extract from the organic Feteasca Neagra, which attached in the second stage of clustering. Several differences are underlined by the graphical representation of SPSS classification of the analyzed samples concerning the hydroalcoholic extracts from seeds. Only the extracts from the grape varieties Merlot, Feteasca Neagra, and Muscat Hamburg cultivated in the conventional system, respectively; Muscat Hamburg cultivated in the organic system proved to have common characteristics in terms of phytochemical content, antioxidant, and antibacterial activity. The extract obtained from the seeds of the Pinot Noir variety cultivated in the conventional system also joined this group in the second stage of clusterization. The seeds from the organic varieties of Merlot and Feteasca Neagra constituted the source of extracts with the same behavior related to variables of interest, clustering in the third stage, and connecting after that with the cluster of the other extracts obtained from seeds, regardless of the system of management. The hydroalcoholic extract from seeds of the Pinot Noir variety cultivated in the organic system was noticed through distinct characteristics to all the other 15 samples analyzed, an aspect that could be exploited in future research.

## 3. Discussion

In recent years, there have been an increasing number of published reports showing the efficient antimicrobial activities of various extracts (Table 5). In this study, favorable antagonistic activities against bacteria from natural habitats, ranging in relatively large limits, were reported for hydroalcoholic extracts obtained from the skins and seeds of grapes cultivated in conventional and organic systems, respectively.

The differences observed can be attributed to climatic conditions [62], with the differences in temperature, sun exposure, and precipitation playing an important role in the accumulation of flavonoids in the skin, anthocyanin accumulation being the most sensitive to these variations. Temperature is one of the main factors influencing the content of flavonoids and their composition in the skin. Previous studies have shown that moderate temperatures and amortization of the fluctuations in diurnal temperatures have improved the accumulation of anthocyanins while high temperatures have reduced this accumulation [63,64].

Variations of the diurnal temperatures mainly affect the composition of hydroxylated anthocyanins, flavonols, and acylated anthocyanins. Solar radiation also plays an important role in the synthesis of flavonoids and their accumulation in the skin, the accumulation of flavonols being more sensitive compared to that of anthocyanins and flavan-3-ols [62,63,65,66]. 

The accumulation of flavonoids in the skin is also influenced by the availability of water, a decrease in this respect causes the accumulation of anthocyanins, flavonols, and 3′, 4′, 5′ substituted compounds in the skin [62].

Different plants have known antimicrobial properties, but the efficacies of grape skins and seeds against bacteria, with a focus on the correlation between a certain chemical compound and its antimicrobial activity, have not been well-documented in the literature. Moreover, the influence of the system of grape management on the extract efficiency is little represented.

The results of the present study showed that the skin of conventional varieties and the seeds of organic varieties have a great potential against Gram-positive bacteria. The hydroalcoholic extracts of these grape varieties exhibited wide zones of inhibition of the tested bacteria growth in the disk diffusion assay. Lower concentrations of extracts are required to inhibit the bacteria growth, as supported by the MIC assay.

The high concentration of bioactive compounds in the plants cultivated in the organic system may be the result of the plants’ exposure to conditions in which the absence of pesticides leads to an increase in the content of natural substances with a protective role [67,68].

Previous literature has reported a broad antibacterial spectrum of grape seed extract, its intensity depending on different factors such as the type of solvent used for extraction, the extract composition, and the concentration of bioactive compounds [38,69].

The hydroalcoholic skin extract of grape (Muscat variety) was found to be efficient against *S. aureus* and *E. faecalis*, with the diameters of the zones of inhibition being 7 mm and 5.9 mm respectively, while a MIC value of 250 mg/mL was determined [70].

Silván et al. [71] demonstrated that the grape seed extract (with a phenolic profile consisting of catechins and proanthocyanidins as major compounds, respectively, flavonols, phenolic acids, and anthocyanins) had a strong capacity to inhibit *Campylobacter* spp. growth. A minimal inhibitory concentration (MIC) of 20 mg/L against *Campylobacter jejuni* was determined. The growth of all the twelve *Campylobacter* strains tested was significantly inhibited by the aqueous extract, at a final concentration of 500 mg GAE/L. It was established that the phenolic acids, catechins, and proanthocyanidins were mainly responsible for the inhibition of *Campylobacter* growth.

Reported effective concentration of grape seed extracts for different microorganisms ranges within a wide interval. Thus, MICs starting from 160 mg/L were effective for *S. aureus* and other Gram-positive microorganisms [72,73], while 4000 mg/L inhibited the growth of *E. coli* [74] and 8000 mg/L the growth of *S. thypimurium* [75].

Different compounds were identified as mainly responsible for the antibacterial activity of grape extracts. One of them is gallic acid [76,77], which was reported to provoke the disintegration of the outer membrane of *Salmonella*, after its permeabilization, based on the chelation of divalent cations [78]. Catechins have been also found to be responsible for inhibiting the growth of some Gram-negative bacteria [79,80], while flavonols have been reported to possess antimicrobial activity against Gram-negative bacteria from the *Enterobacteriaceae* family [81]. The mechanism of action is mainly attributed to cytoplasmic membrane damage and enzymatic inhibition [82].

Gram-negative bacteria have an outer membrane of the cell wall made up of structural lipopolysaccharides, so it is impermeable to lipophilic solutions, unlike Gram-positive bacteria, which do not have this outer membrane. The mentioned morphological difference influences the reaction of bacteria in relationship with the antibacterial agents. In addition, Gram-negative bacteria have several efflux pumps, which prevent the intracellular accumulation of antibacterial agents. This requires the discovery and development of new antibacterial agents that are able to bypass or suppress efflux pumps and that could also restore the antibacterial potential of the generic antibiotics [83].

In other reports, no correlation between the antimicrobial activity of vine leaf hydroalcoholic extracts on Gram-negative and Gram-positive bacteria strains and their contents of flavan-3-ols and flavonols was established [84]. The different degree of response depends on the tested microorganism and the composition in the phenolic compounds of the extracts as well as on the existence of a synergetic effect between the different polyphenolic compounds with an antiradical role and antimicrobial activity [85].

The inhibitory effects of the seed extracts against bacteria are dose-dependent and strain-dependent. The low efficiency found at high concentrations of extract is due to the low solubility in water, with the inhibitory effect of the phenolic compounds from seed extracts being more pronounced in relationship to the Gram-positive strains [86].

Further studies are needed on the phytochemical screening, purification, and quantification of the bioactive components and their antimicrobial activity in relationship with a certain bacterial strain of interest.

## 4. Materials and Methods 

### 4.1. Vineyard Description

**Figure 8:**
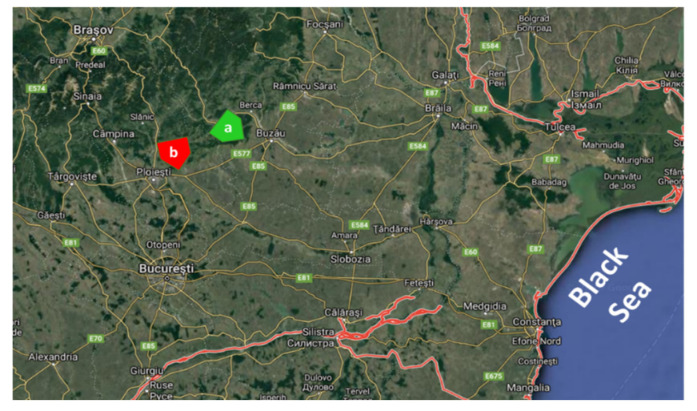
Locations of the studied vineyards in Romania: (**a**) organic (i.e., Franco-Romanian Domains) and (**b**) conventional (i.e., Valea Calugareasca, Prahova County).

The grape materials used in this study were collected from two locations in Romania, one from organic management (Sahateni, Buzau County) and the second from conventional management (Valea Calugareasca, Prahova County). Four red grape varieties were collected from each of these locations as follows: Feteasca Neagra, Pinot Noir, Merlot, and Muscat Hamburg. The organic vineyard (Franco-Romanian Domains, Figure 8) covers an area of 45 hectares with south–southeast exposure and is representative of red wine varieties. The relief is fragmented with slopes of different altitudes and crossed by numerous valleys. The level curves delimiting the grape cultures start from 125 m in the plain area up to 250 m on the hills. The slopes are generally smooth, in the range of 5% to 20%. The climate in the area is continental, characterized by significant temperature differences between day and night, between summer and winter, and from one day to another. These thermal amplitudes contribute to a full expression of these noble grape varieties and in developing a high aging potential. The climate here is also characterized by long and sunny summers, and this favors a good ripening of grapes. This region is open and ventilated due to winds, a favorable condition for drying the soil and plants.

Grape varieties selected for the present study were found in the vineyard on different surfaces as follows: Feteasca Neagra, 6 ha; Pinot Noir, 15.5 ha; Merlot, 2 ha; and Muscat Hamburg, 0.1 ha. Plantations with these four vine varieties were established in 2000, and the following planting distances were applied: 2.2 m between rows, 1.0 m between vines per row, and thus a density of 4450 vines/ha.

The conventional vineyard (Figure 8) that provided samples for the present study was established in 1967 as the Valea Calugareasca Research and Development Institute for Viticulture and is located in Prahova County, 12 km east of Ploiesti, Romania.

The climate in this region is temperate continental, the average annual temperature is 11.3 °C, and the annual rainfall is 642 mm. During vegetation, useful temperatures are a total 3411 degrees, sunshine of 1520 h, and a sum of precipitation of 395 mm. The enoclimatic aptitude index in the region has a value of 4786, corresponding to a very good oenological potential.

### 4.2. Preparation of Grape Extracts

For the current study, grape samples were collected at full maturity, in the autumn of 2019 (i.e., middle of September); after harvesting, the studied grapes (four varieties from each type of culture, organic/conventional) were separated in three fractions: skin, seeds, and pulp. The skin and the seeds were dried for 48 h at 40 °C and were the subject of the present study. 

Hydroalcoholic extracts of studied grape varieties were obtained from skins and seeds, after a previous step of drying in the oven at 40 °C for 48 h. Two extraction methods were applied, classical maceration at room temperature, and ultrasound assisted extraction. For both methods, a weighted amount of approximately 2 g of dried skin was placed in a covered laboratory flask, and a measured volume of 50 mL solvent at room temperature was added. For classical extraction (i.e., maceration), the mixture was kept under magnetic stirring for 3 h, then centrifuged after 21 h, and the total contact time was 24 h. The same procedure was followed for the skin and seeds samples from all studied grape varieties. For the ultrasound assisted method, the total contact time was also 24 h, with the first 30 min maintained in the ultrasound field of 45 kHz. For both methods of extraction, after fulfilling the total contact time, a centrifugation step at 1000 rpm for 10 min followed, and a final filtration was performed (Whatmann no. 4 paper). The clear solution may be used for further analytical measurements of phytochemical properties.

According to preliminary tests, the experiments performed to evaluate the antimicrobial effect of the grape extracts needed more concentrated solutions. Thus, for these experiments, extracts were prepared by weighing 1.2 g of dry weight from the grape fraction (skin or seed, from the respective variety and vineyard), followed by contact with 10 mL of sterile solvent. The total contacting time was the same at 24 h (at room temperature) as well as the initial activation time of 3 h with magnetic stirring for maceration, and 30 min for ultrasound extraction. All the lab-vessels and tools used were sterile, and manipulation was performed under UV light for all these experiments.

### 4.3. Phytochemical Characterization of Grape Extracts

All experimental determinations in this study were performed using chemical reagents of analytical grade. Deionized water (with conductivity at 25 °C below 0.5 μS cm^−1^) was used as the solvent and wash solution. Sodium nitrite (99% purity), aluminum chloride (99% purity), sodium hydroxide (purity >97%, and sodium carbonate <1%) were purchased from Merck Millipore (MiliporeSigma, Burlington, MA, USA); ethanol of analytical grade was purchased from a local producer, Chimopar SA (SC Chimopar Trading SRL, Bucharest, Romania). Quercetin hydrate with 95% purity was purchased from Sigma-Aldrich (MiliporeSigma, Burlington, MA, USA).

Evaluation of antioxidant activity (AA) was performed by using the spectrophotometric method with the formation of the phosphomolybdenic complex compound, adapted for the sample’s used matrices [87]. Ascorbic acid (Figure 1) was used as the standard, and all results were expressed in mg ascorbic acid per gram of sample. 

Total phenolic content (TPC) was estimated using gallic acid (GAE) as the standard reference. The Folin–Ciocalteu reagent reacts with phenolic compounds and non-phenolic reducing substances, also (including ascorbic acid) forming chemical species that can be detected by UV-VIS spectroscopy. The GAE is a well-known standard for the appreciation of TPC in the grape extracts, being representative for the highlighting of phenolic acids from them [6]; readings were performed at 765 nm. The interaction with the gallic acid of the Folin–Ciocalteu reagent is equivalent to most other phenolic compounds from grape extracts.

The total flavonoid content (TFC) was estimated based on the formation of Al^3+^-flavonoid complexes whose absorbance was measured at 510 nm. For the evaluation of TFC, quercetin was used as a standard for the calibration curve, and all the sample results were expressed as mg quercetin per gram of sample.

### 4.4. Test Microorganisms

Eight strains from the Culture Collection of Bacteria of the Institute of Multidisciplinary Research for Science and Technology from Valahia University of Targoviste (in progress) were tested and screened for their ability to resist antimicrobial compounds from grape extracts (skin and seeds, respectively). The strains were chosen to cover different habitats and genres. The fungal strains isolated proved to be relatively resistant to the tested extracts, leading to very small values of the diameters of the inhibition zones or to a lack of these ones.

The bacterial strains included in the study were isolated and characterized by classical microbiological techniques. The isolation of the pure cultures of bacteria was carried out from different habitats (foods of animal origin and plant-based foods, air) after successive replications, so avoiding contamination of the microorganisms used as the test. Agarized meat broth (pH = 6.8 ± 0.2) was used for growth and incubation was achieved at 37 ± 0.2 °C.

Macroscopic and microscopic characterization of colonies was done according to the Bergey’s Manual of Systematic Bacteriology description. The pure cultures of bacteria isolates were maintained on broth agar medium at 4 °C. This analysis indicated that the isolated strains belonged to genus mentioned in Table 2. Eleven strains of bacteria were isolated, of which eight were used in the study. The test microorganisms used for primary in vitro antibacterial screening of the grape extracts are summarized in Table 6.

Bacterial cultures were used to prepare the inoculum for antimicrobial testing by picking a colony from 24-h-old plates, suspended in an appropriate medium, and grown aerobically at 37 °C for 24 h. The size of the inoculum was standardized on a spectrophotometric basis by means of the optical density at the wavelength of 600 nm (OD600 = 0.2–0.4).

### 4.5. Determination of the Antibacterial Activity

The standardized technique of disks impregnated with extracts was used, this method being recommended by the NCCLS (National Committee for Clinical Laboratory Standardization).

A volume (20–50 µL) of fresh bacterial culture with OD600 between 0.2 and 0.4 was spread on Petri plates with agar meat broth. On the inoculated plates were placed sterile 6 mm paper disks, previously impregnated for 1 h in the grape extracts (skin and seeds extracts, respectively, prepared according to the described protocol to avoid contamination). The disks were placed approximately 15 mm from the periphery of the plate and 30 mm from each other, respectively. The plates were incubated at 37 ± 0.2 °C for 48 h. The extracts with antibacterial activity showed a clear area (halo) around the colony due to the inhibition of the growth of the tested bacteria strain. The interpretation of the results was made by the diameters of the inhibition zones according to the Clinical Laboratory Standards Institute (CLSI) standard.

The determinations were performed in triplicate. The results, expressed as average values obtained by the arithmetic mean of the diameters corresponding to the three tests, were directly proportional to the sensitivity of the tested microorganism (the inhibition zone of the bacterial growth is even wider when the chemical compounds from the grape extracts are more active).

### 4.6. Determination of Minimum Inhibitory Concentration (MIC)

The value of the minimum inhibitory concentration (MIC) of the grape extracts was determined by selecting the lowest concentration of each extract, which completely inhibited the growth of the tested microorganism, an aspect detected by the unaided eye. To establish the growth end points, control samples (without plant extracts) were prepared. The standardized inoculum of each tested strain (inoculum obtained by transferring 3–5 colonies in the nutrient broth with a sterilized loop) was seeded in a discontinuous gradient of concentrations of each grape extract in tubes with nutrient broth. After incubation at 37 ± 0.2 °C for 48 h, the MIC value was read by macroscopic observation of the tubes. In the first tubes, with high concentrations of extract, the growth of the culture was not visible, the bacteria being destroyed or inhibited. The extract concentration corresponding to the tube with the lowest concentration, which completely inhibited the growth of the organism, represented the MIC value (µg/mL) for the respective type of extract.

### 4.7. Statistical Analysis of Data

The experiments were performed in triplicate. The results were expressed as mean values ± standard deviation (SD). Data analysis was performed using Statistical Package for the Social Science v24.0 software for MS Windows. The analysis of variance (ANOVA) at the 5% level of significance was carried out to evaluate the significance of differences in the means of various groups. Multiple linear regression, bivariate correlations of data (on the basis of the Pearson coefficients), and the SPSS classification through hierarchical cluster analysis were carried out to assess the potential relationship between the chemical compounds, their antimicrobial activity, vineyard management, the grape variety, and the anatomic part from which the hydroalcoholic extracts were obtained, respectively.

## 5. Conclusions

The antimicrobial activity of the grapes’ hydroalcoholic extracts was determined based on the observation and quantification of the growth of some strains of bacteria isolated from natural environments, brought into contact with agents with antimicrobial potential from the skin and seeds, respectively.

Most of the tested grape extracts (from skin and seeds) showed a significant antimicrobial activity against the spoilage bacteria selected from natural environments, depending on the anatomical part and the species of microorganism.

The hydroalcoholic extracts obtained from the skin of the grape varieties cultivated in the conventional system showed a significant antibacterial activity compared to extracts obtained from the same varieties, but grown in an organic system. The hydroalcoholic extracts obtained from the seeds of the grape varieties from the organic system showed a broader spectrum of antibacterial activity, compared to the ones cultivated in a conventional system.

The highest values of the antibacterial activity were recorded in the case of the hydroalcoholic extracts from the seeds of organic varieties of Muscat Hamburg and Merlot, respectively, and Pinot Noir (conventional system of management).

Research on the antimicrobial activity of the grape extracts, correlated with their antioxidant activity, provides the needed scientific basis for the isolation and purification of bioactive chemical compounds from grapes, compounds that could be used in therapeutics, in order to solve the phenomenon of antibiotic resistance as well as for skin diseases, mainly to solve the problems of atopic dermatitis; in the food industry, in order to replace some chemical preservatives, preventing food spoilage and the human health hazards associated with chemical applications; and in the food industry to design nutraceuticals and products with health benefits. It is necessary to continue this research to test the microbial strains of interest for a certain application of the grape extracts.

## Figures and Tables

**Table 1:** TPC, TFC, and AA values for grape skin and seeds hydroalcoholic extracts.

Grape Variety	Vineyard Type	Total Phenolic Content [mg GAE/g]		Total Flavonoids Content [mg Quercetin/g]		Antioxidant Activity [mg Ascorbic Acid/g]	
		Grape Berry Tissue					
		*Skin*	*Seeds*	*Skin*	*Seeds*	*Skin*	*Seeds*
Merlot	Organic	55.69 ± 3.18 ^ab^	146.80 ± 6.53 ^b^	43.94 ± 3.84	172.19 ± 9.67	21.22 ± 1.39 ^a^	355.77 ± 9.57
	Conventional	15.82 ± 0.50 ^ab^	47.38 ± 0.90 ^b^	51.89 ± 3.44	120.69 ± 8.53	24.22 ± 1.92 ^a^	143.2 ± 7.04
Feteasca Neagra	Organic	71.98 ± 4.04 ^ab^	150.92 ± 4.87 ^b^	87.72 ± 5.95	158.36 ± 11.10	23.99 ± 2.16 ^a^	286.58 ± 10.47
	Conventional	22.17 ± 0.58 ^ab^	64.48 ± 1.36 ^b^	47.02 ± 2.87	122.14 ± 7.18	23.82 ± 2.62 ^a^	157.07 ± 9.31
Pinot Noir	Organic	47.04 ± 1.87 ^ab^	169.53 ± 7.32 ^b^	26.28 ± 1.46	388.25 ± 10.72	15.98 ± 1.53 ^a^	312.84 ± 12.81
	Conventional	20.64 ± 1.53 ^ab^	77.05 ± 2.76 ^b^	15.79 ± 1.51	135.13 ± 5.68	19.36 ± 1.99 ^a^	209.59 ± 11.38
Muscat Hamburg	Organic	20.41 ± 1.26 ^ab^	52.78 ± 1.90 ^b^	49.23 ± 3.07	123.58 ± 8.66	26.55 ± 2.35 ^a^	135.77 ± 8.14
	Conventional	19.94 ± 1.73 ^ab^	73.53 ± 1.37 ^b^	43.41 ± 3.63	131.76 ± 6.70	26.32 ± 2.09 ^a^	164.5 ± 6.45

^a^ significant difference (*p* ≤ 0.05) among grapes’ varieties; ^b^ significant difference (*p* ≤ 0.05) among vineyard management, concerning the phytochemical characteristics of extracts (one-way ANOVA, Tukey test).

**Table 2:** Measure of association between the phytochemical characteristics and the system of grape management, respectively, on their anatomic part.

Association	Eta Squared
Antioxidant activity * Management system	0.051
Flavonoids * Management system	0.075
Phenolics * Management system	0.241
Antioxidant activity * Anatomic part	0.752
Flavonoids * Anatomic part	0.502
Phenolics * Anatomic part	0.445

**Table 3:** Regression analysis between the compounds of interest.

Equation of Regression	R Square
Total phenolic content (TPC) = 70.529−8.688 * variety −49.065 * vineyard management +61.299 * anatomic part	0.72
Total flavonoid content (TFC) = 14.027−1.927 * variety −52.094 * vineyard management +118.972 * anatomic Part	0.56
Antioxidant activity = −25.377−17.632 * variety −59.569 * vineyard management +189.74 * anatomic Part	0.82

**Table 4:** Measure of association between the antimicrobial activity of extracts and the system of grape management, respectively, on their anatomic part.

Association	Eta Squared	Association	Eta Squared
CCB1 * management system	0.122	CCB1 * anatomic part	0.030
CCB3 * management system	0.000	CCB3 * anatomic part	0.010
CCB4 * management system	0.019	CCB4 * anatomic part	0.171
CCB5 * management system	0.003	CCB5 * anatomic part	0.030
CCB6 * management system	0.001	CCB6 * anatomic part	0.001
CCB7 * management system	0.015	CCB7 * anatomic part	0.029
CCB10 * management system	0.005	CCB10 * anatomic part	0.164

**Table 5:** Antimicrobial properties of the compounds extracted.

Type	Compounds	Antimicrobial Activity	Reference
Polyphenols	Phlorotannins	Alteration of the cell membrane and cell destruction of *S. aureus*, *S*. *pneumonia* and *P. aeruginosa*	[40]
	Phlorotannins	Alteration of the cell membrane, cytoplasm’s leakage and cell destruction of *V. parahaemolyticus*	[41]
	Phlorofucofuroeckol	Cell membrane damage and suppression of genes related to methicillin resistance in *S. aureus*	[42]
	Bromophenols	Downregulation of pathogenic genes of *P. gingivalis*	[43]
	Dieckol	Alteration of cell integrity and metabolism of *T. rubrum*	[44]
	Phlorotannins	Alterations of the cell wall composition, increased mitochondrial respiration. Inhibition of the formation of the germ tube of *C. albicans*	[45]
	Phlorotannins	Inhibition of the enzyme neuraminidase of the Influenza A virus	[46]
	Polyphenolic rich extracts	Inhibition of the viral particle	[47]
Polysaccharides	Depolymerized fucoidans	Interaction with protein of the cell membrane and cellular rupture of *E. coli* and S. *aureus*	[48]
	Fucoidan	Inhibition of dental plaque bacteria and foodborne pathogens.	[49]
	Laminarin rich extracts	Inhibition of *S. aureus*, *L. monocytogenes*, *E. coli* and *S. typhimurium*.	[50]
	Water soluble polysaccharide extracts	Inhibition of *F*. *oxy*sp*orium* Inhibition of *C. albicans* and *M. phaseli*	[51]
	Sulfated polysaccharides	Obstruction of herpes simplex virus type 1 and 2 attachment to the cells	[52]
		Interference with fusion between HIV infected cells. Inhibition of the viral enzyme reverse transcriptase	
		Inhibition of dengue virus by interaction with the glycoprotein of the viral envelop	
Proteins & peptides	Lectins	Inhibition of several Gram-negative bacteria by interaction with compounds of the cell wall	[53]
	Lectins	Inhibition of *T. rubrum* and *C. lindemuthianum*	
	Lectins	Antiviral effects against HIV, Hepatitis C virus and SARS-CoV by preventing the entry in the host cells	[54]
Fatty acids	Bioactive fraction	Perforation of the cell wall of *S. aureus* and *K. neumoniae*, cytoplasmic leakage and cell death	[55]
	Bioactive fraction	Rupture of cell membrane of *Vibrio* spp and *A*. *hydrophila*	[56]
	Bioactive fraction	Fatty acids could be involved in the inhibition *S. aureus*, *E. coli* and *P. vulgaris*	[57]
	Bioactive fraction	Inhibition of *C. cladosporioides* and *C.* s*phaerospermum* by disrupting the cell membrane	[58]
	Sulfoquinovosyldia-cylglycerol	Antiviral effects against HSV type 2 by disturbing the initial stages of the viral life cycle	[59]
Pigments	Fucoxanthin	Inhibition of *L. monocytogenes*	[60]
	Fucoxanthin	Inhibition of several pathogenic bacteria by increasing cell membrane permeability, leakage of cytoplasm and inhibition of nucleic acid	[61]

**Table 6:** Bacterial strains used in the study.

No.	Specie/Code	Source
1	*Lactococcus* sp./CCB1	Wheat
2	*Bacillus* sp. T3/CCB3	Nuts
3	*Bacillus* sp./CCB4	Seeds
4	*Lactobacillus* sp./CCB5	Meat products
5	*Streptococcus* sp./CCB6	Dairy products
6	*Leuconostoc* sp./CCB7	Vegetables
7	*Micrococcus* sp./CCB10	Air (vineyard)
8	*Bacillus* sp./CCB11	Wheat

## References

[1] Eurostat—Database. https://ec.europa.eu/eurostat/web/agriculture/data.

[2] Leal C., Gouvinhas I., Santos R.A., Rosa E., Silva A.M., Saavedra M.J., Barros A.I.R.N.A. (2020). Potential application of grape (*Vitis vinifera* L.) stem extracts in the cosmetic and pharmaceutical industries: Valorization of a by-product. Ind. Crop. Prod..

[3] Anastasiadi M., Chorianopoulos N.G., Nychas G.J.E., Karoutounian S.A. (2009). Antilisterial activities of polyphenol-rich extracts of grapes and vinification byproducts. J. Agric. Food Chem..

[4] Coderoni S., Perito M.A. (2020). Sustainable consumption in the circular economy. An analysis of consumers’ purchase intentions for waste-to-value food. J. Clean. Prod..

[5] Mattos G.N., Tonon R.V., Furtado A.A.L., Cabral L.M.C. (2017). Grape by-product extracts against microbial proliferation and lipid oxidation: A review. J. Sci. Food Agric..

[6] Melo P.S., Massarioli A.P., Denny C., Dos Santos L.F., Franchin M., Pereira G.E., Vieira T.M.F., Rosalen P.L., de Alencar S.M. (2015). Winery by-products: Extraction optimization, phenolic composition and cytotoxic evaluation to act as a new source of scavenging of reactive oxygen species. Food Chem..

[7] Leal C., Santos R.A., Pinto R., Queiroz M., Rodrigues M., José Saavedra M., Barros A., Gouvinhas I. (2020). Recovery of bioactive compounds from white grape (*Vitis vinifera* L.) stems as potential antimicrobial agents for human health. Saudi J. Biol. Sci..

[8] Friedman M. (2014). Antibacterial, Antiviral, and Antifungal Properties of Wine and Winery Byproducts in Relation to Their Flavonoid Content. J. Agric. Food Chem..

[9] Garcia-Lomillo J., Gonzalez-SanJose M.L. (2017). Applications of Wine Pomace in the Food Industry: Approaches and Functions. Compr. Rev. Food Sci. Food Saf..

[10] Business Wire. https://www.businesswire.com/news/home/20200228005268/en/Grape-Seed-Oil-Market-2020-2024-Increasing-Application.

[11] Pisoschi A.M., Pop A., Georgescu C., Turcuş V., Olah K., Mathe E. (2017). An overview of natural antimicrobials role in food. Eur. J. Med. Chem..

[12] Queiroz M., Oppolzer D., Gouvinhas I., Silva A.M., Barros A.I.R.N.A., Domínguez-Perles R. (2017). New grape stems’ isolated phenolic compounds modulate reactive oxygen species, glutathione, and lipid peroxidation in vitro: Combined formulations with vitamins C and E. Fitoterapia.

[13] Das R., Bhattacharjee C., Jaiswal A.K. (2020). Chapter 43—Grapes. Nutritional Composition and Antioxidant Properties of Fruits and Vegetables.

[14] Peixoto C.M., Ines M., Alves M.J., Calhelha R.C. (2018). Grape pomace as a source of phenolic compounds and diverse bioactive properties. Food Chem..

[15] Działo M., Mierziak J., Korzun U., Preisner M., Szopa J., Kulma A. (2016). The Potential of Plant Phenolics in Prevention and Therapy of Skin Disorders. Int. J. Mol. Sci..

[16] Nicolescu C.M., Bumbac M., Olteanu R.L., Alecu (Holban) G.C., Boboaca-Mihaescu D.N., Necula C., Radulescu C. (2019). Influence Of Extraction Method On Chemical Composition From Red Grapes Skin Extract. J. Sci. Arts.

[17] Radulescu C., Olteanu R.L., Stihi C., Florescu M., Stirbescu R.M., Stanescu S.G., Nicolescu C.M., Bumbac M. (2020). Chemometrics based-vibrational spectroscopy for *Juglandis semen* extracts investigation. J. Chemom..

[18] Alecu (Holban) G.C., Olteanu R.L., Radulescu C., Stirbescu R.M., Necula C., Boboaca-Mihaescu D.N. (2020). Characterization of red grapes skin extracts using vibrational spectroscopy and chemometrics. J. Sci. Arts.

[19] Radulescu C., Olteanu R.L., Stihi C., Florescu M., Lazurca D., Dulama I.D., Stirbescu R.M., Teodorescu S. (2019). Chemometric assessment of spectroscopic techniques and antioxidant activity for *Hippophae rhamnoides* L. extracts obtained by different isolation methods. Anal. Lett..

[20] Buruleanu L., Radulescu C., Georgescu A.A., Nicolescu C.M., Olteanu R.L., Dulama I.D., Stanescu G.S. (2019). Chemometric Assessment of the Interactions between the Metal Contents, Antioxidant Activity, Total Phenolics, and Flavonoids in Mushrooms. Anal. Lett..

[21] David M., Serban A., Radulescu C., Danet A.F., Florescu M. (2019). Bioelectrochemical evaluation of plant extracts with antioxidant capacity using gold nanozyme-based sensor. Bioelectrochemistry.

[22] Draelos Z.D. (2019). Quality skincare on a budget, summer sun protection. Dermatol. Times.

[23] Reed R. (1962). The definition of “cosmeceutical”. J. Soc. Cosmet. Chem..

[24] Mathes S.H., Ruffner H., Graf-Hausner U. (2014). The use of skin models in drug development. Adv. Drug Deliv. Rev..

[25] Benitez J.M., Montans F.J. (2017). The mechanical behavior of skin: Structures and models for the finite element analysis. Comput. Struct..

[26] Azimi H., Fallah-Tafti M., Khalshur A.A., Abdollahi M. (2012). A review of phytotherapy of acne vulgaris: Perspective of new pharmacological treatments. Fitoterapia.

[27] Kligman A. (2005). The Future of Cosmeceuticals: An Interview with Albert Kligman, MD, PhD. Dermatol. Surg..

[28] Faria-Silva C., Ascenso A., Costa A.M., Marto J., Calvarheiro M., Ribeiro H.M., Simoes S. (2020). Feeding the skin: A new trend in food and cosmetics convergence. Trends Food Sci. Technol..

[29] Anunciato T.P., Rocha-Filho P. (2012). Carotenoids and polyphenols in nutricosmetics, nutraceuticals, and cosmeceuticals. J. Cosmet. Dermatol..

[30] Nunes M.A., Rodrigues F., Oliveira M.B.P.P., Galanakis C.M. (2017). Grape Processing By-Products as Active Ingredients for Cosmetic Proposes. Handbook of Grape Processing By-Products. Sustainable Solutions.

[31] Santos A.C., Rodrigues D., Sequeira J.A.D., Pereira I., Simoes A., Costa D., Peixoto D., Costa G., Veiga F. (2019). Nanotechnological breakthroughs in the development of topical phytocompounds-based formulations. Int. J. Pharm..

[32] Zucchinelli M., D’Ammaro D., Giubilato E., Zabeo A., Criscione P., Pizzol L., Cohen Y., Tarolli P., Lamastra L., Marinello F. (2020). Use of multiple indicators to compare sustainability performance of organic vs. conventional vineyard management. Sci. Tot. Environ..

[33] Milićević T., Aničić Urošević M., Relić D., Jovanović G., Nikolić D., Vergel K., Popović A. (2020). Environmental pollution influence to soil–plant–air system in organic vineyard: Bioavailability, environmental, and health risk assessment. Environ. Sci. Pollut. Res..

[34] Uzman D., Leyer I., Reineke A., Entling M.H. (2020). Differential effects of semi-natural habitats and organic management on spiders in viticultural landscapes. Agric. Ecosyst. Environ..

[35] Xu W., Bo L., Wang C., Kong X. (2020). Organic management of grape affects yeast succession and wine sensory quality during spontaneous fermentation. LWT Food Sci. Technol..

[36] Zahedipour P., Asghari M., Abdollahi B., Alizadeh M., Danesh Y.R. (2019). A comparative study on quality attributes and physiological responses of organic and conventionally grown table grapes during cold storage. Sci. Hortic..

[37] Cravero M.C. (2020). Organic and biodynamic wines quality and characteristics: A review. Food Chemistry.

[38] Furiga A., Lonvaud-Funel A., Badet C. (2009). In vitro study of antioxidant capacity and antibacterial activity on oral anaerobes of a grape seed extract. Food Chemistry..

[39] Ivanišová E., Terentjeva M., Kántor A., Frančáková H., Kačániová M. (2019). Phytochemical and Antioxidant Profile of Different Varieties of Grape from the Small Carpathians Wine Region of Slovakia. Erwerbs Obstbau.

[40] Bogolitsyn K., Dobrodeeva L., Druzhinina A., Ovchinnikov D., Parshina A., Shulgina E. (2019). Biological activity of a polyphenolic complex of Arctic brown algae. J. Appl. Phycol..

[41] Wei Y., Liu Q., Xu C., Yu J., Zhao L., Guo Q. (2016). Damage to the Membrane Permeability and Cell Death of *Vibrio parahaemolyticus* Caused by Phlorotannins with Low Molecular Weight from Sargassum thunbergii. J. Aquat. Food Prod. Technol..

[42] Eom S.H., Lee D.S., Jung Y.J., Park J.H., Choi J.I., Yim M.J., Jeon J.M., Kim H.W., Son K.T., Je J.Y. (2014). The mechanism of antibacterial activity of phlorofucofuroeckol-A against methicillin-resistant *Staphylococcus aureus*. Appl. Microbiol. Biotechnol..

[43] Cherian C., Jannet Vennila J., Sharan L. (2019). Marine bromophenols as an effective inhibitor of virulent proteins (peptidyl arginine deiminase, gingipain R and hemagglutinin A) in *Porphyromas gingivalis*. Arch. Oral Biol..

[44] Lee M.H., Lee K.B., Oh S.M., Lee B.H., Chee H.Y. (2010). Antifungal activities of dieckol isolated from the marine brown alga *Ecklonia cava* against *Trichophyton rubrum*. J. Appl. Biol. Chem..

[45] Lopes G., Pinto E., Andrade P.B., Valentão P. (2013). Antifungal Activity of Phlorotannins against Dermatophytes and Yeasts: Approaches to the Mechanism of Action and Influence on *Candida albicans* Virulence Factor. PLoS ONE.

[46] Ryu Y.B., Jeong H.J., Yoon S.Y., Park J.Y., Kim Y.M., Park S.J., Rho M.C., Kim S.J., Lee W.S. (2011). Influenza virus neuraminidase inhibitory activity of phlorotannins from the edible brown alga *Ecklonia cava*. J. Agric. Food Chem..

[47] Morán-Santibañez K., Peña-Hernáncez M.A., Cruz-Suárez L.E., Ricque-Marie D., Skouta R., Vasquez A.H., Rodríguez-Padilla C., Trejo-Avila L.M. (2018). Virucidal and Synergistic Activity of Polyphenol-Rich Extracts of Seaweeds against Measles Virus. Viruses.

[48] Liu M., Liu Y., Cao M., Liu G., Chen Q., Sun L., Chen H. (2017). Antibacterial activity and mechanisms of depolymerized fucoidans isolated from *Laminaria japonica*. Carbohydr. Polym..

[49] Iun J.J., Jung M., Jeong I., Yamazaki K., Kawai Y., Kim B.-M. (2018). Antimicrobial and Antibiofilm Activities of Sulfated Polysaccharides from Marine Algae against Dental. Mar. Drugs.

[50] Kadam S.U., O’Donnell C.P., Rai D.K., Hossain M.B., Burgess C.M., Walsh D., Tiwari B.K. (2015). Laminarin from Irish brown seaweeds *Ascophyllum nodosum* and *Laminaria hyperborea*: Ultrasound assisted extraction, characterization and bioactivity. Mar. Drugs.

[51] Zeid A.H.A., Aboutabl E.A., Sleem A.A., El-Rafie H.M. (2014). Water soluble polysaccharides extracted from *Pterocladia capillacea* and *Dictyopteris membranacea* and their biological activities. Carbohydr. Polym..

[52] Siahaan E.A., Pangestuti R., Kim S.-K. (2018). Seaweeds: Valuable Ingredients for the Pharmaceutical Industries.

[53] Singh R.S., Walia A.K. (2018). Lectins from red algae and their biomedical potential. J. Appl. Phycol..

[54] Cheung R.C.F., Wong J.H., Pan W., Chan Y.S., Yin C., Dan X., Ng T.B. (2015). Marine lectins and their medicinal applications. Appl. Microbiol. Biotechnol..

[55] El Shafay S.M., Ali S.S., El-Sheekh M.M. (2016). Antimicrobial activity of some seaweeds species from Red sea, against multidrug resistant bacteria. Egypt. J. Aquat. Res..

[56] Kasanah N., Amelia W., Mukminin A., Isnansetyo T., Isnansetyo A. (2019). Antibacterial activity of Indonesian red algae *Gracilaria edulis* against bacterial fish pathogens and characterization of active fractions. Nat. Prod. Res..

[57] Anjali K.P., Sangeetha B.M., Devi G., Raghunathan R., Dutta S. (2019). Bioprospecting of seaweeds (*Ulva lactuca* and *Stoechospermum marginatum*): The compound characterization and functional applications in medicine-a comparative study. J. Photochem. Photobiol. B Biol..

[58] de Felício R., de Albuquerque S., Young M.C.M., Yokoya N.S., Debonsi H.M. (2010). Trypanocidal, leishmanicidal and antifungal potential from marine red alga Bostrychia tenella J. Agardh (Rhodomelaceae, Ceramiales). J. Pharm. Biomed. Anal..

[59] Wang H., Li Y., Shen W., Rui W., Ma X., Cen Y. (2007). Antiviral activity of a sulfoquinovosyldiacylglycerol (SQDG) compound isolated from the green alga *Caulerpa racemosa*. Bot. Mar..

[60] Rajauria G., Abu-ghannam N. (2013). Isolation and Partial Characterization of Bioactive Fucoxanthin from *Himanthalia elongata* Brown Seaweed: A TLC-Based Approach. Int. J. Anal. Chem..

[61] Karpinski T.M., Adamczak A. (2019). Fucoxanthin—An Antibacterial Carotenoid. Antioxidants.

[62] Zhu L., Huang Y., Xu C., Lu J., Wang Y. (2017). The growing season impacts the accumulation and composition of flavonoids in grape skins in two-crop-a-year viticulture. J. Food Sci. Technol..

[63] Spayd S.E., Tarara J.M., Mee D.L., Ferguson J.C. (2002). Separation of sunlight and temperature effects on the composition of *Vitis vinifera* cv. Merlot berries. Am. J. Enol. Vitic..

[64] Cohen S.D., Tarara J.M., Kennedy J.A. (2008). Assessing the impact of temperature of grape phenolic metabolism. Anal. Chim. Acta.

[65] Downey M.O., Dokoozlian N.K., Krstic M.P. (2006). Cultural practice and environmental impacts on the flavonoid composition of grape and wine: A review of recent research. Am. J. Enol. Vitic..

[66] Haselgrove L., Botting D., van Heeswijck R., Hoj P.B., Dry P.R., Ford C., Iland P.G. (2000). Canopy microclimate and berry composition: The effect of bunch exposure in the phenolic composition of *Vitis vinifera L.* cv. Shiraz grape berries. Aust. J. Grape Wine Res..

[67] Bunea C.I., Pop N., Babes A.C., Mantea C., Dulf F.V., Bunea A. (2012). Carotenoids, total polyphenols, and antioxidant activity of grapes (*Vitis vinifera*) cultivated in organic and conventional systems. Chem. Cent. J..

[68] Winter C.K., Davis S.F. (2006). Organic Foods. J. Food Sci..

[69] Chedea V.S., Braicu C., Chirila F., Ober C., Socaciu C. (2011). Antibacterial action of an aqueous grape seed polyphenolic extract. Afr. J. Biotechnol..

[70] Nirmala G., Narendhirakannan R.T. (2011). In vitro antioxidant and antimicrobial activities of grapes (*Vitis vinifera L*) seed and skin extracts—Muscat variety. Int. J. Pharm. Sci..

[71] Silván J.M., Mingo E., Hidalgo M., de Pascual-Teresa S., Carrascosa A.V., Martinez-Rodriguez A.J. (2013). Antibacterial activity of a grape seed extract and its fractions against *Campylobacter spp*. Food Control.

[72] Baydar N.G., Sagdic O., Ozkan G., Cetin S. (2006). Determination of antibacterial effects and total phenolic contents of grape (*Vitis vinifera L.*) seed extracts. Int. J. Food Sci. Technol..

[73] Yigit D., Yigit N., Mavi A., Yildirim A., Güleryüz M. (2009). Antioxidant and antimicrobial activities of methanol and water extracts of fruits, leaves and seeds of *Vitis vinifera L.* cv. Karaerik. Asian J. Chem..

[74] Over K.F., Hettiarachchy N., Johnson M.G., Davis B. (2009). Effect of organic acids and plant extracts on Escherichia coli O157:H7, Listeria monocytogenes, and *Salmonella Typhimurium* in broth culture model and chicken meat systems. J. Food Sci..

[75] Ahn J., Grün I.U., Mustapha A. (2004). Antimicrobial and antioxidant activities of natural extracts in vitro and in ground beef. J. Food Prot..

[76] Rajamanickam K., Yang J., Sakharkar M.K. (2018). Gallic Acid Potentiates the Antimicrobial Activity of Tulathromycin against Two Key Bovine Respiratory Disease (BRD) Causing-Pathogens. Front Pharmacol..

[77] Naz S., Siddiqi R., Ahmad S., Rasool S., Sayeed S.A. (2007). Antibacterial activity directed isolation of compounds from *Punica granatum*. J. Food Sci..

[78] Nohynek L.J., Alakomi H.L., Kahkonen M., Heinone M., Helander I.M., Oksman-Caldentey K.M., Puupponen-Pimia R. (2006). Berry phenolics: Antimicrobial properties and mechanisms of action against severe human pathogens. Nutr. Cancer.

[79] Cowan M.M. (1999). Plant products as antimicrobials agents. Clin. Microbiol. Rev..

[80] Ankolekar C., Johnson D., Pinto M.D., Johnson K., Labbe R., Shetty K. (2011). Inhibitory potential of tea polyphenolics and influence of extraction time against *Helicobacter pylori* and lack of inhibition of beneficial lactic acid bacteria. J. Med. Food.

[81] Yao W.R., Wang H.Y., Wang S.T., Sun S.L., Zhou J., Luan Y.Y. (2011). Assessment of the antibacterial activity and the antidiarrheal function of flavonoids from bayberry fruit. J. Agric. Food Chem..

[82] Cushnie T.P.T., Lamb A.J. (2011). Recent advances in understanding the antibacterial properties of flavonoids. Int. J. Antimicrob. Agents.

[83] Piddock L.J. (2006). Clinically relevant chromosomally encoded multidrug resistance efflux pumps in bacteria. Clin. Microbiol. Rev..

[84] Abramovič H., Terpinc P., Generalić I., Skroza D., Klancnik A., Katalinic V., Možina S.S. (2012). Antioxidant and antimicrobial activity of extracts obtained from rosemary (*Rosmarinus officinalis*) and vine (*Vitis vinifera*) leaves. Croat. J. Food Sci. Technol..

[85] El Darra N., Tannous J., Bou Mounsef P., Palge J., Vaghi J., Voroliev E., Louka N., Maroun R.S. (2012). A Comparative Study on Antiradical and Antimicrobial Properties of Red Grapes Extracts Obtained from Different *Vitis vinifera* Varieties. Food Nutr. Sci..

[86] Soto M.L., Falque E., Dominguez H. (2015). Relevance of Natural Phenolics from Grape and Derivative Products in the Formulation of Cosmetics. Cosmetics.

[87] Prieto P., Pineda M., Aguilar M. (1999). Spectrophotometric Quantitation of Antioxidant Capacity through the Formation of a Phosphomolybdenum Complex: Specific Application to the Determination of Vitamin E. Anal. Biochem..

